# Supplementary material for: Analysis of Epidemiological and Evolutionary Characteristics of Seasonal Influenza Viruses in Shenzhen City from 2018 to 2024
Source: Viruses. 2025 May 30;17(6):798. doi: 10.3390/v17060798 (PMC12197669; doi:10.3390/v17060798)
Supplement: Supplementary file 1 [file viruses-17-00798-s001.zip › viruses-3572731-supplementary.pdf]

Table S1. Primer sequences for seasonal influenza virus detection by RT-PCR

Subtype	Prime name	Primer sequence
H1N1pdm09	H1-F	5‘-CAA CCA AA ATG A AGG CAA AAC TACT
	H1-R	5‘-CTCATGATTCTGAAATCCTAATG
	N1-F	5'-GTT TAA AAT GAA TCC AAA CCA AAA GAT
	N1-R	5'-GGC AAC TCA GCA CCG TCT GGC CAA G
H3N2	H3-F	5’-CTATTAACCATGAAGACTATCATTG
	H3-R	5'-TTTAATTAATGCACTCAAATGC
	N2-F	5'-GTAAAGATGAATCCAAATCAAAAGAT
	N2-R	5'-TTCTAAAATTGCGAAAGCTTAT
B/Victoria	B/HA-F	5’-GCA GAG CAT TTT CTA ATA TCC ACAA
	B/HA-R	5’-GCACTACAATAAAGGAAAATACA
	B/NA-F	5’-CTG AAG CAA ATA GGC CAA AAA TGA AC
	B/NA-R	5’-AGGAACAAAGGGTTTAGAACAGACT

Table S2. Information and GISAID numbers of isolated strains and other reference viruses

Subtype	Isolate-Name	Segment_IDs	Originating lab
H1N1pdm09	A/shenzhen/1/2019	HA: EPI3915507<br>NA: EPI3915508	Shenzhen Center for Disease Control and Prevention
	A/shenzhen/2/2019	HA: EPI3916211<br>NA: EPI3916210	Shenzhen Center for Disease Control and Prevention
	A/shenzhen/3/2019	HA: EPI3916213<br>NA: EPI3916212	Shenzhen Center for Disease Control and Prevention
	A/shenzhen/4/2019	HA: EPI3916215<br>NA: EPI3916214	Shenzhen Center for Disease Control and Prevention
	A/shenzhen/5/2019	HA: EPI3916217<br>NA: EPI3916216	Shenzhen Center for Disease Control and Prevention
	A/shenzhen/6/2019	HA: EPI3916219<br>NA: EPI3916218	Shenzhen Center for Disease Control and Prevention
	A/shenzhen/7/2019	HA: EPI3916221<br>NA: EPI3916220	Shenzhen Center for Disease Control and Prevention
	A/shenzhen/8/2019	HA: EPI3916223<br>NA: EPI3916222	Shenzhen Center for Disease Control and Prevention
	A/shenzhen/9/2019	HA: EPI3916225<br>NA: EPI3916224	Shenzhen Center for Disease Control and Prevention
	A/shenzhen/10/2019	HA: EPI3916227<br>NA: EPI3916226	Shenzhen Center for Disease Control and Prevention
	A/shenzhen/11/2019	HA: EPI3916229<br>NA: EPI3916228	Shenzhen Center for Disease Control and Prevention
	A/shenzhen/12/2019	HA: EPI3916231<br>NA: EPI3916230	Shenzhen Center for Disease Control and Prevention
	A/shenzhen/13/2019	HA: EPI3916233<br>NA: EPI3916232	Shenzhen Center for Disease Control and Prevention

H1N1pdm09	A/shenzhen/14/2019	HA: EPI3916235<br>NA: EPI3916234	Shenzhen Center for Disease Control and Prevention
	A/shenzhen/15/2019	HA: EPI3916237<br>NA: EPI3916236	Shenzhen Center for Disease Control and Prevention
	A/Michigan/45/2015	HA: EPI1381204<br>NA: EPI1381203	WHO Chinese National Influenza Center
	A/Belgium/8414/2019	HA: EPI1444883<br>NA: EPI1444885	
	A/California/52/2019	HA: EPI1382841<br>NA: EPI1382840	California Department of Health Services
	A/Italy/8933/2019	HA: EPI1501082<br>NA: EPI1501250	
	A/USA/PV02768/2019	HA: EPI3173878<br>NA: EPI3173873	CEIRR, Influenza Data Processing and Communication Center
	A/Michigan/UOMMH21718/2019	HA: EPI3113657<br>NA: EPI3113655	University of Michigan Clinical Microbiology Laboratory
	A/Brisbane/02/2018	HA: EPI1504919<br>NA: EPI1504918	WHO Chinese National Influenza Center
	A/Hawaii/56/2018	HA: EPI1345472<br>NA: EPI1345471	State of Hawaii Department of Health
	A/Iowa/56/2019	HA: EPI1726564<br>NA: EPI1726563	Iowa State Hygienic Laboratory
	A/Hawaii/70/2019	HA: EPI1617983<br>NA: EPI1617982	State of Hawaii Department of Health
	A/Guangdong-Maonan/SWL1536/2019	HA: EPI1716630<br>NA: EPI1716632	Guangdong Provincial Center for Disease Control and Prevention
	A/Beijing/24/2019	HA: EPI1788916<br>NA: EPI1788964	

H1N1pdm09	A/Pennsylvania/04/2019	HA: EPI1361488<br>NA: EPI1361487	Pennsylvania Department of Health
	A/Baltimore/R0496/2019	HA: EPI1893900<br>NA: EPI1893898	
	A/California/7810/2019	HA: EPI1520459<br>NA: EPI1520621	
	A/shenzhen/1/2023	HA: EPI3916239<br>NA: EPI3916238	Shenzhen center for disease control and prevention
	A/shenzhen/2/2023	HA: EPI3916241<br>NA: EPI3916240	Shenzhen center for disease control and prevention
	A/shenzhen/3/2023	HA: EPI3916243<br>NA: EPI3916242	Shenzhen center for disease control and prevention
	A/shenzhen/4/2023	HA: EPI3916245<br>NA: EPI3916244	Shenzhen center for disease control and prevention
	A/shenzhen/5/2023	HA: EPI3916247<br>NA: EPI3916246	Shenzhen center for disease control and prevention
	A/shenzhen/6/2023	HA: EPI3916249<br>NA: EPI3916248	Shenzhen center for disease control and prevention
	A/shenzhen/7/2023	HA: EPI3916251<br>NA: EPI3916250	Shenzhen center for disease control and prevention
	A/shenzhen/8/2023	HA: EPI3916253<br>NA: EPI3916252	Shenzhen center for disease control and prevention
	A/shenzhen/9/2023	HA: EPI3916255<br>NA: EPI3916254	Shenzhen center for disease control and prevention
	A/shenzhen/10/2023	HA: EPI3916257<br>NA: EPI3916256	Shenzhen center for disease control and prevention
	A/shenzhen/11/2023	HA: EPI3916259<br>NA: EPI3916258	Shenzhen center for disease control and prevention
	A/shenzhen/12/2023	HA: EPI3916261<br>NA: EPI3916260	Shenzhen center for disease control and prevention

	A/shenzhen/13/2023	HA: EPI3916263<br>NA: EPI3916262	Shenzhen center for disease control and prevention
	A/shenzhen/14/2023	HA: EPI3916265<br>NA: EPI3916264	Shenzhen center for disease control and prevention
	A/shenzhen/15/2023	HA: EPI3916267<br>NA: EPI3916266	Shenzhen center for disease control and prevention
	A/shenzhen/16/2023	HA: EPI3916269<br>NA: EPI3916268	Shenzhen center for disease control and prevention
	A/shenzhen/17/2023	HA: EPI3916271<br>NA: EPI3916270	Shenzhen center for disease control and prevention
	A/shenzhen/18/2023	HA: EPI3916273<br>NA: EPI3916272	Shenzhen center for disease control and prevention
**H1N1pdm09**	A/shenzhen/19/2023	HA: EPI3916275<br>NA: EPI3916274	Shenzhen center for disease control and prevention
	A/shenzhen/20/2023	HA: EPI3916277<br>NA: EPI3916276	Shenzhen center for disease control and prevention
	A/shenzhen/21/2023	HA: EPI3916279<br>NA: EPI3916278	Shenzhen center for disease control and prevention
	A/Wisconsin/588/2019	HA: EPI3571883<br>NA: EPI3571881	Centers for Disease Control and Prevention
	A/Victoria/2570/2019	HA: EPI2436681<br>NA: EPI2436683	Seqirus Pty Ltd (CSL Group)
	A/Wisconsin/67/2022	HA: EPI3997954<br>NA: EPI3997952	Centers for Disease Control and Prevention
	A/Victoria/4897/2022	HA: EPI3592841<br>NA: EPI3592839	WHO Collaborating Centre for Reference and Research on Influenza
	A/Ohio/09/2023	HA: EPI2781201<br>NA: EPI2781199	Ohio Department of Health Laboratories

H1N1pdm09	A/Hawaii/28/2023	HA: EPI2677696<br>NA: EPI2677688	State of Hawaii Department of Health
	A/Nevada/50/2023	HA: EPI3056270<br>NA: EPI3056265	Southern Nevada Public Health Lab
	A/Sichuan-Shunqing/SWL11211/2023	HA: EPI2973146<br>NA: EPI2973145	WHO Chinese National Influenza Center
	A/Guangdong-Futian/SWL11589/2023	HA: EPI2822344<br>NA: EPI2822343	WHO Chinese National Influenza Center
	A/Anhui-Qiaocheng/SWL11278/2023	HA: EPI2959468<br>NA: EPI2959467	WHO Chinese National Influenza Center
	A/Hubei-Xiantao/SWL2916/2023	HA: EPI2822353<br>NA: EPI2822352	WHO Chinese National Influenza Center
	A/Heilongjiang-Taoshan/SWL1484/2023	HA: EPI2668072<br>NA: EPI2668071	WHO Chinese National Influenza Center
	A/Texas/204/2023	HA: EPI3727830<br>NA: EPI3727829	Baylor Scott and White Health
	A/Washington/100/2023	HA: EPI3560834<br>NA: EPI3560831	Marshfield Clinic Research Foundation
	A/shenzhen/1/2024	HA: EPI3916281<br>NA: EPI3916280	Shenzhen center for disease control and prevention
	A/shenzhen/2/2024	HA: EPI3916283<br>NA: EPI3916282	Shenzhen center for disease control and prevention
	A/shenzhen/3/2024	HA: EPI3916285<br>NA: EPI3916284	Shenzhen center for disease control and prevention
	A/shenzhen/4/2024	HA: EPI3916287<br>NA: EPI3916286	Shenzhen center for disease control and prevention
	A/shenzhen/5/2024	HA: EPI3916289<br>NA: EPI3916288	Shenzhen center for disease control and prevention

	A/shenzhen/6/2024	HA: EPI3916291<br>NA: EPI3916290	Shenzhen center for disease control and prevention
	A/shenzhen/7/2024	HA: EPI3916293<br>NA: EPI3916292	Shenzhen center for disease control and prevention
	A/shenzhen/8/2024	HA: EPI3916295<br>NA: EPI3916294	Shenzhen center for disease control and prevention
	A/shenzhen/9/2024	HA: EPI3916297<br>NA: EPI3916296	Shenzhen center for disease control and prevention
	A/shenzhen/10/2024	HA: EPI3916299<br>NA: EPI3916298	Shenzhen center for disease control and prevention
	A/shenzhen/11/2024	HA: EPI3916301<br>NA: EPI3916300	Shenzhen center for disease control and prevention
**H1N1pdm09**	A/shenzhen/12/2024	HA: EPI391630<br>NA: EPI3916302	Shenzhen center for disease control and prevention
	A/shenzhen/13/2024	HA: EPI3916305<br>NA: EPI3916304	Shenzhen center for disease control and prevention
	A/shenzhen/14/2024	HA: EPI3916307<br>NA: EPI3916306	Shenzhen center for disease control and prevention
	A/shenzhen/15/2024	HA: EPI3916309<br>NA: EPI3916308	Shenzhen center for disease control and prevention
	A/shenzhen/16/2024	HA: EPI3916311<br>NA: EPI3916310	Shenzhen center for disease control and prevention
	A/shenzhen/17/2024	HA: EPI3916313<br>NA: EPI3916312	Shenzhen center for disease control and prevention
	A/shenzhen/18/2024	HA: EPI3916315<br>NA: EPI3916314	Shenzhen center for disease control and prevention
	A/shenzhen/19/2024	HA: EPI3916317<br>NA: EPI3916316	Shenzhen center for disease control and prevention

H1N1pdm09	A/shenzhen/20/2024	HA: EPI3916319<br>NA: EPI3916318	Shenzhen center for disease control and prevention
	A/shenzhen/21/2024	HA: EPI3916321<br>NA: EPI3916320	Shenzhen center for disease control and prevention
	A/shenzhen/22/2024	HA: EPI3916323<br>NA: EPI3916322	Shenzhen center for disease control and prevention
	A/Sichuan-Guanghan/SWL2704/2024	HA: EPI3684559<br>NA: EPI3684558	WHO Chinese National Influenza Center
	A/Shanghai-Putuo/SWL1666/2024	HA: EPI3684568<br>NA: EPI3684567	WHO Chinese National Influenza Center
	A/Guangdong-Dongwanbendi/SWL2511/2024	HA: EPI3467141<br>NA: EPI3467140	WHO Chinese National Influenza Center
	A/Maryland/70/2024	HA: EPI3760094<br>NA: EPI3760093	Maryland Department of Health and Mental Hygiene
	A/California/LACPHL-INF00802/2024	HA: EPI3767930<br>NA: EPI3767928	Los Angeles County Public Health Laboratories, Lab Microbial Pathogen Submission Group
	A/Florida/21/2024	HA: EPI3760102<br>NA: EPI3760101	Florida Department of Health-Tampa
	A/Norway/05416/2024	HA: EPI3492654<br>NA: EPI3492653	Norwegian Institute of Public Health
H3N2	A/shenzhen/1/2019	HA: EPI3917480<br>NA: EPI3917479	Shenzhen center for disease control and prevention
	A/shenzhen/2/2019	HA: EPI3917482<br>NA: EPI3917481	Shenzhen center for disease control and prevention

H3N2	A/shenzhen/3/2019	HA: EPI3917484<br>NA: EPI3917483	Shenzhen center for disease control and prevention
	A/shenzhen/4/2019	HA: EPI3916215<br>NA: EPI3916214	Shenzhen center for disease control and prevention
	A/shenzhen/5/2019	HA: EPI3917486<br>NA: EPI3917485	Shenzhen center for disease control and prevention
	A/shenzhen/6/2019	HA: EPI3917488<br>NA: EPI3917487	Shenzhen center for disease control and prevention
	A/shenzhen/7/2019	HA: EPI3917490<br>NA: EPI3917489	Shenzhen center for disease control and prevention
	A/shenzhen/8/2019	HA: EPI3916223<br>NA: EPI3916222	Shenzhen center for disease control and prevention
	A/shenzhen/9/2019	HA: EPI3917492<br>NA: EPI3917491	Shenzhen center for disease control and prevention
	A/shenzhen/10/2019	HA: EPI3916227<br>NA: EPI3916226	Shenzhen center for disease control and prevention
	A/shenzhen/11/2019	HA: EPI3917494<br>NA: EPI3917493	Shenzhen center for disease control and prevention
	A/Arizona/05/2019	HA: EPI1421236<br>NA: EPI1421235	Arizona Department of Health Services
	A/Georgia/29/2019	HA: EPI1495751<br>NA: EPI1495750	Georgia Public Health Laboratory
	A/Keelung/R0020/2019	HA: EPI1893931<br>NA: EPI1893936	
	A/Hawaii/20/2019	HA: EPI1429631<br>NA: EPI1429630	State of Hawaii Department of Health
	A/South Australia/34/2019	HA: EPI1607117<br>NA: EPI1607116	WHO Collaborating Centre for Reference and Research on Influenza
	A/Kansas/14/2017	HA: EPI1440506<br>NA: EPI1440505	New York Medical College

H3N2	A/Singapore/INFIMH-16-0019/2016	HA: EPI1858150<br>NA: EPI1858151	Centers for Disease Control and Prevention
	A/Hong Kong/45/2019	HA: EPI1691930<br>NA: EPI1691929	WHO Chinese National Influenza Center
	A/Darwin/726/2019	HA: EPI1658695<br>NA: EPI1658694	Royal Darwin Hospital
	A/Minnesota/41/2019	HA: EPI1726517<br>NA: EPI1726516	Minnesota Department of Health
	A/Wenzhou/1951/2019	HA： EPI2147004<br>NA: EPI2147019	
	A/Delaware/39/2019	HA: EPI1602887<br>NA: EPI1602886	Delaware Public Health Lab
	A/shenzhen/1/2022	HA: EPI3917496<br>NA: EPI3917495	Shenzhen center for disease control and prevention
	A/shenzhen/2/2022	HA: EPI3917498<br>NA: EPI3917497	Shenzhen center for disease control and prevention
	A/shenzhen/3/2022	HA: EPI3917500<br>NA: EPI3917499	Shenzhen center for disease control and prevention
	A/shenzhen/4/2022	HA: EPI3917502<br>NA: EPI3917501	Shenzhen center for disease control and prevention
	A/shenzhen/5/2022	HA: EPI3917504<br>NA: EPI3917503	Shenzhen center for disease control and prevention
	A/shenzhen/6/2022	HA: EPI3917506<br>NA: EPI3917505	Shenzhen center for disease control and prevention
	A/shenzhen/7/2022	HA: EPI3917508<br>NA: EPI3917507	Shenzhen center for disease control and prevention
	A/shenzhen/8/2022	HA: EPI3917510<br>NA: EPI3917509	Shenzhen center for disease control and prevention
	A/shenzhen/9/2022	HA: EPI3917512<br>NA: EPI3917511	Shenzhen center for disease control and prevention

H3N2	A/shenzhen/10/2022	HA: EPI3917514<br>NA: EPI3917513	Shenzhen center for disease control and prevention
	A/shenzhen/11/2022	HA: EPI3917516<br>NA: EPI3917515	Shenzhen center for disease control and prevention
	A/shenzhen/12/2022	HA: EPI3917518<br>NA: EPI3917517	Shenzhen center for disease control and prevention
	A/shenzhen/13/2022	HA: EPI3917520<br>NA: EPI3917519	Shenzhen center for disease control and prevention
	A/shenzhen/14/2022	HA: EPI3917522<br>NA: EPI3917521	Shenzhen center for disease control and prevention
	A/shenzhen/15/2022	HA: EPI3917524<br>NA: EPI3917523	Shenzhen center for disease control and prevention
	A/shenzhen/16/2022	HA: EPI3917526<br>NA: EPI3917525	Shenzhen center for disease control and prevention
	A/shenzhen/17/2022	HA: EPI3917528<br>NA: EPI3917527	Shenzhen center for disease control and prevention
	A/shenzhen/18/2022	HA: EPI3917530<br>NA: EPI3917529	Shenzhen center for disease control and prevention
	A/shenzhen/19/2022	HA: EPI3917532<br>NA: EPI3917531	Shenzhen center for disease control and prevention
	A/shenzhen/20/2022	HA: EPI3917534<br>NA: EPI3917533	Shenzhen center for disease control and prevention
	A/shenzhen/21/2022	HA: EPI3917536<br>NA: EPI3917535	Shenzhen center for disease control and prevention
	A/shenzhen/22/2022	HA: EPI3917538<br>NA: EPI3917537	Shenzhen center for disease control and prevention
	A/shenzhen/23/2022	HA: EPI3917540<br>NA: EPI3917539	Shenzhen center for disease control and prevention

	A/shenzhen/24/2022	HA: EPI3917542<br>NA: EPI3917541	Shenzhen center for disease control and prevention
	A/Shanghai/CN1796C2/2022	HA: EPI3588892<br>NA: EPI3588890	Shanghai Municipal Center for Disease Control and Prevention
	A/Washington/47/2022	HA: EPI2225793<br>NA: EPI2225792	Washington State Public Health Laboratory
	A/Shanghai/FX1540C2/2022	HA: EPI3588588<br>NA: EPI3588586	Shanghai Municipal Center for Disease Control and Prevention
	A/Shanghai/HK1678C1/2022	HA: EPI3590148<br>NA: EPI3590146	Shanghai Municipal Center for Disease Control and Prevention
	A/Cambodia/e0826360/2020	HA: EPI1837753<br>NA: EPI1837752	Institute Pasteur du Cambodia
**H3N2**	A/shenzhen/1/2023	HA: EPI3917544<br>NA: EPI3917543	Shenzhen center for disease control and prevention
	A/shenzhen/2/2023	HA: EPI3916241<br>NA: EPI3916240	Shenzhen center for disease control and prevention
	A/shenzhen/3/2023	HA: EPI3916243<br>NA: EPI3916242	Shenzhen center for disease control and prevention
	A/shenzhen/4/2023	HA: EPI3917546<br>NA: EPI3917545	Shenzhen center for disease control and prevention
	A/shenzhen/5/2023	HA: EPI3917548<br>NA: EPI3917547	Shenzhen center for disease control and prevention
	A/shenzhen/6/2023	HA: EPI3917550<br>NA: EPI3917549	Shenzhen center for disease control and prevention
	A/shenzhen/7/2023	HA: EPI3917552<br>NA: EPI3917551	Shenzhen center for disease control and prevention
	A/shenzhen/8/2023	HA: EPI3917554<br>NA: EPI3917553	Shenzhen center for disease control and prevention

H3N2	A/shenzhen/9/2023	HA: EPI3916255<br>NA: EPI3916254	Shenzhen center for disease control and prevention
	A/shenzhen/10/2023	HA: EPI3916257<br>NA: EPI3916256	Shenzhen center for disease control and prevention
	A/shenzhen/11/2023	HA: EPI3917556<br>NA: EPI3917555	Shenzhen center for disease control and prevention
	A/shenzhen/12/2023	HA: EPI3916261<br>NA: EPI3916260	Shenzhen center for disease control and prevention
	A/shenzhen/13/2023	HA: EPI3916263<br>NA: EPI3916262	Shenzhen center for disease control and prevention
	A/shenzhen/14/2023	HA: EPI3917558<br>NA: EPI3917557	Shenzhen center for disease control and prevention
	A/shenzhen/15/2023	HA: EPI3916267<br>NA: EPI3916266	Shenzhen center for disease control and prevention
	A/shenzhen/16/2023	HA: EPI3916269<br>NA: EPI3916268	Shenzhen center for disease control and prevention
	A/shenzhen/17/2023	HA: EPI3916271<br>NA: EPI3916270	Shenzhen center for disease control and prevention
	A/shenzhen/18/2023	HA: EPI3916273<br>NA: EPI3916272	Shenzhen center for disease control and prevention
	A/shenzhen/19/2023	HA: EPI3916275<br>NA: EPI3916274	Shenzhen center for disease control and prevention
	A/shenzhen/20/2023	HA: EPI3916277<br>NA: EPI3916276	Shenzhen center for disease control and prevention
	A/Human/New York City/PV83892/2023	HA: EPI3334009<br>NA: EPI3334007	CEIRS Data Processing and Coordinating Center, Center for Research on Influenza Pathogenesis (CRIP)

	A/Human/New York City/PV85324/2022	HA: EPI3333578<br>NA: EPI3333576	CRIP
	A/YAMANASHI/23063/2023	HA: EPI2630450<br>NA: EPI2630449	Yamanashi Institute for Public Health
	A/Norway/07235/2023	HA: EPI2619090<br>NA: EPI2619089	CRIP
	A/Singapore/NUH0012/2023	HA: EPI2614806<br>NA: EPI2614805	Ministry of Health, Singapore
	A/Massachusetts/18/2022	HA: EPI3654415<br>NA: EPI3654413	Centers for Disease Control and Prevention
	A/Thailand/8/2022	HA: EPI2178977<br>NA: EPI2178976	WHO National Influenza Centre, National Institute of Medical Research (NIMR)
**H3N2**	A/Wisconsin/27/2023	HA: EPI2498594<br>NA: EPI2498592	Wisconsin State Laboratory of Hygiene
	A/Washington/20/2023	HA: EPI2498958<br>NA: EPI2498956	Washington State Public Health Laboratory
	A/Minnesota/17/2023	HA: EPI2464190<br>NA: EPI2464188	Minnesota Department of Health
	A/shenzhen/1/2024	HA: EPI3916281<br>NA: EPI3916280	Shenzhen center for disease control and prevention
	A/shenzhen/2/2024	HA: EPI3916283<br>NA: EPI3916282	Shenzhen center for disease control and prevention
	A/shenzhen/3/2024	HA: EPI3916285<br>NA: EPI3916284	Shenzhen center for disease control and prevention
	A/shenzhen/4/2024	HA: EPI3917560<br>NA: EPI3917559	Shenzhen center for disease control and prevention
	A/shenzhen/5/2024	HA: EPI3916289<br>NA: EPI3916288	Shenzhen center for disease control and prevention
	A/shenzhen/6/2024	HA: EPI3916291<br>NA: EPI3916290	Shenzhen center for disease control and prevention

	A/shenzhen/7/2024	HA: EPI3916293<br>NA: EPI3916292	Shenzhen center for disease control and prevention
	A/shenzhen/8/2024	HA: EPI3917562<br>NA: EPI3917561	Shenzhen center for disease control and prevention
	A/shenzhen/9/2024	HA: EPI3916297<br>NA: EPI3916296	Shenzhen center for disease control and prevention
	A/shenzhen/10/2024	HA: EPI3916299<br>NA: EPI3916298	Shenzhen center for disease control and prevention
	A/shenzhen/11/2024	HA: EPI3916301<br>NA: EPI3916300	Shenzhen center for disease control and prevention
	A/shenzhen/12/2024	HA: EPI3916303<br>NA: EPI3916302	Shenzhen center for disease control and prevention
	A/shenzhen/13/2024	HA: EPI3916305<br>NA: EPI3916304	Shenzhen center for disease control and prevention
**H3N2**	A/shenzhen/14/2024	HA: EPI3916307<br>NA: EPI3916306	Shenzhen center for disease control and prevention
	A/shenzhen/15/2024	HA: EPI3916309<br>NA: EPI3916308	Shenzhen center for disease control and prevention
	A/shenzhen/16/2024	HA: EPI3916311<br>NA: EPI3916310	Shenzhen center for disease control and prevention
	A/shenzhen/17/2024	HA: EPI3916313<br>NA: EPI3916312	Shenzhen center for disease control and prevention
	A/shenzhen/18/2024	HA: EPI3916315<br>NA: EPI3916314	Shenzhen center for disease control and prevention
	A/shenzhen/19/2024	HA: EPI3916317<br>NA: EPI3916316	Shenzhen center for disease control and prevention
	A/shenzhen/20/2024	HA: EPI3916319<br>NA: EPI3916318	Shenzhen center for disease control and prevention

H3N2	A/shenzhen/21/2024	HA: EPI3916321<br>NA: EPI3916320	Shenzhen center for disease control and prevention
	A/shenzhen/22/2024	HA: EPI3916323<br>NA: EPI3916322	Shenzhen center for disease control and prevention
	A/shenzhen/23/2024	HA: EPI3917564<br>NA: EPI3917563	Shenzhen center for disease control and prevention
	A/Massachusetts/ISC-1046/2024	HA: EPI3188875<br>NA: EPI3188873	Massachusetts Department of Public Health
	A/Hong Kong/EPI0467/2024	HA: EPI3360892<br>NA: EPI3360890	University of Hong Kong, School of Public Health, WHO Collaborating Centre for Infectious Disease Epidemiology and Control
	A/Idaho/21/2024	HA: EPI3396663<br>NA: EPI3396662	State of Idaho Bureau of Laboratories
	A/Croatia/10136RV/2023	HA: EPI3625269<br>NA: EPI3625270	Croatian Institue of Public Health
	A/Missouri/119/2024	HA: EPI3531103<br>NA: EPI3531102	Centers for Disease Control and Prevention
	A/Nevada/165/2024	HA: EPI3520587<br>NA: EPI3520586	Southern Nevada Public Health Lab
B/Victoria	B/shenzhen/1/2019	HA: EPI3916325<br>NA: EPI3916324	Shenzhen center for disease control and prevention
	B/shenzhen/2/2019	HA: EPI3916327<br>NA: EPI3916326	Shenzhen center for disease control and prevention
	B/shenzhen/3/2019	HA: EPI3916329<br>NA: EPI3916328	Shenzhen center for disease control and prevention
	B/shenzhen/4/2019	HA: EPI3916331<br>NA: EPI3916330	Shenzhen center for disease control and prevention

	B/shenzhen/5/2019	HA: EPI3916333<br>NA: EPI3916332	Shenzhen center for disease control and prevention
	B/shenzhen/6/2019	HA: EPI3916335<br>NA: EPI3916334	Shenzhen center for disease control and prevention
	B/shenzhen/7/2019	HA: EPI3916337<br>NA: EPI3916336	Shenzhen center for disease control and prevention
	B/shenzhen/8/2019	HA: EPI3916339<br>NA: EPI3916338	Shenzhen center for disease control and prevention
	B/shenzhen/9/2019	HA: EPI3916341<br>NA: EPI3916340	Shenzhen center for disease control and prevention
	B/shenzhen/10/2019	HA: EPI3916343<br>NA: EPI3916342	Shenzhen center for disease control and prevention
	B/shenzhen/11/2019	HA: EPI3916345<br>NA: EPI3916344	Shenzhen center for disease control and prevention
**B/Victoria**	B/shenzhen/12/2019	HA: EPI3916347<br>NA: EPI3916346	Shenzhen center for disease control and prevention
	B/shenzhen/13/2019	HA: EPI3916349<br>NA: EPI3916348	Shenzhen center for disease control and prevention
	B/shenzhen/14/2019	HA: EPI3916351<br>NA: EPI3916350	Shenzhen center for disease control and prevention
	B/shenzhen/15/2019	HA: EPI3916353<br>NA: EPI3916352	Shenzhen center for disease control and prevention
	B/shenzhen/16/2019	HA: EPI3916355<br>NA: EPI3916354	Shenzhen center for disease control and prevention
	B/shenzhen/17/2019	HA: EPI3916357<br>NA: EPI3916356	Shenzhen center for disease control and prevention
	B/Pennsylvania/08/2020	HA: EPI1714953<br>NA: EPI1714952	Pennsylvania Department of Health

B/Victoria	B/Florida/45/2019	HA: EPI1602771<br>NA: EPI1602770	Florida Department of Health-Jacksonville
	B/Washington/02/2019	HA: EPI1368874<br>NA: EPI1368872	Washington State Public Health Laboratory
	B/Louisiana/56/2019	HA: EPI1638077<br>NA: EPI1638076	Louisiana Department of Health and Hospitals
	B/Michigan/11/2019	HA: EPI1422052<br>NA: EPI1422051	Michigan Department of Community Health
	B/Bolivia/663/2019	HA: EPI1603880<br>NA: EPI1603879	CENETROP
	B/Colorado/06/2017	HA: EPI969380<br>NA: EPI969379	Colorado Department of Health Lab
	B/Canberra/5/2019	HA: EPI1673871<br>NA: EPI1673870	Canberra Hospital
	B/Louisiana/16/2019	HA: EPI1439144<br>NA: EPI1439143	Louisiana Department of Health and Hospitals
	B/shenzhen/1/2021	HA: EPI3916359<br>NA: EPI3916358	Shenzhen center for disease control and prevention
	B/shenzhen/2/2021	HA: EPI3916361<br>NA: EPI3916360	Shenzhen center for disease control and prevention
	B/shenzhen/3/2021	HA: EPI3916523<br>NA: EPI3916522	Shenzhen center for disease control and prevention
	B/shenzhen/4/2021	HA: EPI3916363<br>NA: EPI3916362	Shenzhen center for disease control and prevention
	B/shenzhen/5/2021	HA: EPI3916365<br>NA: EPI3916364	Shenzhen center for disease control and prevention
	B/shenzhen/6/2021	HA: EPI3916367<br>NA: EPI3916366	Shenzhen center for disease control and prevention
	B/shenzhen/7/2021	HA: EPI3916369<br>NA: EPI3916368	Shenzhen center for disease control and prevention

	B/shenzhen/8/2021	HA: EPI3916371<br>NA: EPI3916370	Shenzhen center for disease control and prevention
	B/shenzhen/9/2021	HA: EPI3916373<br>NA: EPI3916372	Shenzhen center for disease control and prevention
	B/shenzhen/10/2021	HA: EPI3916375<br>NA: EPI3916374	Shenzhen center for disease control and prevention
	B/shenzhen/11/2021	HA: EPI3916377<br>NA: EPI3916376	Shenzhen center for disease control and prevention
	B/shenzhen/12/2021	HA: EPI3916379<br>NA: EPI3916378	Shenzhen center for disease control and prevention
	B/shenzhen/13/2021	HA: EPI3916381<br>NA: EPI3916380	Shenzhen center for disease control and prevention
**B/Victoria**	B/shenzhen/14/2021	HA: EPI3916383<br>NA: EPI3916382	Shenzhen center for disease control and prevention
	B/shenzhen/15/2021	HA: EPI3916385<br>NA: EPI3916384	Shenzhen center for disease control and prevention
	B/shenzhen/16/2021	HA: EPI3916387<br>NA: EPI3916386	Shenzhen center for disease control and prevention
	B/shenzhen/17/2021	HA: EPI3916389<br>NA: EPI3916388	Shenzhen center for disease control and prevention
	B/shenzhen/18/2021	HA: EPI3916391<br>NA: EPI3916390	Shenzhen center for disease control and prevention
	B/shenzhen/19/2021	HA: EPI3916393<br>NA: EPI3916392	Shenzhen center for disease control and prevention
	B/shenzhen/20/2021	HA: EPI3916395<br>NA: EPI3916394	Shenzhen center for disease control and prevention
	B/shenzhen/21/2021	HA: EPI3916397<br>NA: EPI3916396	Shenzhen center for disease control and prevention

B/Victoria	B/shenzhen/22/2021	HA: EPI3916399<br>NA: EPI3916398	Shenzhen center for disease control and prevention
	B/shenzhen/23/2021	HA: EPI3916401<br>NA: EPI3916400	Shenzhen center for disease control and prevention
	B/shenzhen/24/2021	HA: EPI3916403<br>NA: EPI3916402	Shenzhen center for disease control and prevention
	B/shenzhen/25/2021	HA: EPI3916405<br>NA: EPI3916404	Shenzhen center for disease control and prevention
	B/shenzhen/30/2021	HA: EPI3916407<br>NA: EPI3916406	Shenzhen center for disease control and prevention
	B/Guangdong/41/2021	HA: EPI3453126<br>NA: EPI3453125	Sun Yat-Sen University of Medical Sciences
	B/Guangdong/129/2021	HA: EPI3453190<br>NA: EPI3453189	Sun Yat-Sen University of Medical Sciences
	B/shenzhen/1/2022	HA: EPI3916409<br>NA: EPI3916408	Shenzhen center for disease control and prevention
	B/shenzhen/2/2022	HA: EPI3916411<br>NA: EPI3916410	Shenzhen center for disease control and prevention
	B/shenzhen/3/2022	HA: EPI3916413<br>NA: EPI3916412	Shenzhen center for disease control and prevention
	B/shenzhen/4/2022	HA: EPI3916415<br>NA: EPI3916414	Shenzhen center for disease control and prevention
	B/shenzhen/5/2022	HA: EPI3916417<br>NA: EPI3916416	Shenzhen center for disease control and prevention
	B/shenzhen/6/2022	HA: EPI3916419<br>NA: EPI3916418	Shenzhen center for disease control and prevention
	B/shenzhen/7/2022	HA: EPI3916421<br>NA: EPI3916420	Shenzhen center for disease control and prevention

	B/shenzhen/8/2022	HA: EPI3916423<br>NA: EPI3916422	Shenzhen center for disease control and prevention
	B/shenzhen/9/2022	HA: EPI3916425<br>NA: EPI3916424	Shenzhen center for disease control and prevention
	B/shenzhen/10/2022	HA: EPI3916427<br>NA: EPI3916426	Shenzhen center for disease control and prevention
	B/shenzhen/11/2022	HA: EPI3916429<br>NA: EPI3916428	Shenzhen center for disease control and prevention
	B/shenzhen/12/2022	HA: EPI3916431<br>NA: EPI3916430	Shenzhen center for disease control and prevention
	B/Guangdong/533/2022	HA: EPI3453134<br>NA: EPI3453133	Sun Yat-Sen University of Medical Sciences
**B/Victoria**	B/Guangdong/182/2021	HA: EPI3453186<br>NA: EPI3453185	Sun Yat-Sen University of Medical Sciences
	B/Montana/01/2022	HA: EPI2003964<br>NA: EPI2003963	Montana Laboratory Services Bureau
	B/Austria/1359417/202	HA: EPI1845793<br>NA: EPI1845794	University of Vienna
	B/Bhutan/0790/2022	HA: EPI2554497<br>NA: EPI2554492	Royal Centre for Disease Control
	B/shenzhen/1/2023	HA: EPI3916433<br>NA: EPI3916432	Shenzhen center for disease control and prevention
	B/shenzhen/2/2023	HA: EPI3916435<br>NA: EPI3916434	Shenzhen center for disease control and prevention
	B/shenzhen/3/2023	HA: EPI3916437<br>NA: EPI3916436	Shenzhen center for disease control and prevention
	B/shenzhen/4/2023	HA: EPI3916439<br>NA: EPI3916438	Shenzhen center for disease control and prevention
	B/shenzhen/5/2023	HA: EPI3916441<br>NA: EPI3916440	Shenzhen center for disease control and prevention

B/Victoria	B/shenzhen/6/2023	HA: EPI3916443<br>NA: EPI3916442	Shenzhen center for disease control and prevention
	B/shenzhen/7/2023	HA: EPI3916445<br>NA: EPI3916444	Shenzhen center for disease control and prevention
	B/shenzhen/8/2023	HA: EPI3916447<br>NA: EPI3916446	Shenzhen center for disease control and prevention
	B/shenzhen/9/2023	HA: EPI3916449<br>NA: EPI3916448	Shenzhen center for disease control and prevention
	B/shenzhen/10/2023	HA: EPI3916451<br>NA: EPI3916450	Shenzhen center for disease control and prevention
	B/shenzhen/11/2023	HA: EPI3916453<br>NA: EPI3916452	Shenzhen center for disease control and prevention
	B/shenzhen/12/2023	HA: EPI3916455<br>NA: EPI3916454	Shenzhen center for disease control and prevention
	B/shenzhen/13/2023	HA: EPI3916457<br>NA: EPI3916456	Shenzhen center for disease control and prevention
	B/shenzhen/14/2023	HA: EPI3916459<br>NA: EPI3916458	Shenzhen center for disease control and prevention
	B/shenzhen/15/2023	HA: EPI3916461<br>NA: EPI3916460	Shenzhen center for disease control and prevention
	B/shenzhen/16/2023	HA: EPI3916463<br>NA: EPI3916462	Shenzhen center for disease control and prevention
	B/shenzhen/17/2023	HA: EPI3916465<br>NA: EPI3916464	Shenzhen center for disease control and prevention
	B/shenzhen/18/2023	HA: EPI3916467<br>NA: EPI3916466	Shenzhen center for disease control and prevention
	B/shenzhen/19/2023	HA: EPI3916469<br>NA: EPI3916468	Shenzhen center for disease control and prevention

**B/Victoria**	B/shenzhen/20/2023	HA: EPI3916471<br>NA: EPI3916470	Shenzhen center for disease control and prevention
	B/shenzhen/21/2023	HA: EPI3916473<br>NA: EPI3916472	Shenzhen center for disease control and prevention
	B/shenzhen/22/2023	HA: EPI3916475<br>NA: EPI3916474	Shenzhen center for disease control and prevention
	B/shenzhen/23/2023	HA: EPI3916477<br>NA: EPI3916476	Shenzhen center for disease control and prevention
	B/shenzhen/24/2023	HA: EPI3916479<br>NA: EPI3916478	Shenzhen center for disease control and prevention
	B/shenzhen/25/2023	HA: EPI3916481<br>NA: EPI3916480	Shenzhen center for disease control and prevention
	B/shenzhen/1/2024	HA: EPI3916483<br>NA: EPI3916482	Shenzhen center for disease control and prevention
	B/shenzhen/2/2024	HA: EPI3916485<br>NA: EPI3916484	Shenzhen center for disease control and prevention
	B/shenzhen/3/2024	HA: EPI3916487<br>NA: EPI3916486	Shenzhen center for disease control and prevention
	B/shenzhen/4/2024	HA: EPI3916489<br>NA: EPI3916488	Shenzhen center for disease control and prevention
	B/shenzhen/5/2024	HA: EPI3916491<br>NA: EPI3916490	Shenzhen center for disease control and prevention
	B/shenzhen/6/2024	HA: EPI3916493<br>NA: EPI3916492	Shenzhen center for disease control and prevention
	B/shenzhen/9/2024	HA: EPI3916495<br>NA: EPI3916494	Shenzhen center for disease control and prevention
	B/shenzhen/10/2024	HA: EPI3916497<br>NA: EPI3916496	Shenzhen center for disease control and prevention

**B/Victoria**	B/shenzhen/11/2024	HA: EPI3916499<br>NA: EPI3916498	Shenzhen center for disease control and prevention
	B/shenzhen/12/2024	HA: EPI3916501<br>NA: EPI3916500	Shenzhen center for disease control and prevention
	B/shenzhen/13/2024	HA: EPI3916503<br>NA: EPI3916502	Shenzhen center for disease control and prevention
	B/shenzhen/14/2024	HA: EPI3916505<br>NA: EPI3916504	Shenzhen center for disease control and prevention
	B/shenzhen/15/2024	HA: EPI3916507<br>NA: EPI3916506	Shenzhen center for disease control and prevention
	B/shenzhen/19/2024	HA: EPI3916509<br>NA: EPI3916508	Shenzhen center for disease control and prevention
	B/shenzhen/20/2024	HA: EPI3916511<br>NA: EPI3916510	Shenzhen center for disease control and prevention
	B/shenzhen/21/2024	HA: EPI3916513<br>NA: EPI3916512	Shenzhen center for disease control and prevention
	B/shenzhen/22/2024	HA: EPI3916525<br>NA: EPI3916524	Shenzhen center for disease control and prevention
	B/Texas/19/2024	HA: EPI3580511<br>NA: EPI3580510	Texas Department of State Health Services-Laboratory Services
	B/New Jersey/13/2024	HA: EPI3342318<br>NA: EPI3342317	New Jersey Department of Health & Senior Services
	B/Xinjiang-Yizhou/11006/2023	HA: EPI2959237<br>NA: EPI2959236	WHO Chinese National Influenza Center
	B/Michigan/UM-10055359417/2024	HA: EPI3034177<br>NA: EPI3034172	University of Michigan, Lauring Lab, Department of Microbiology and Immunology
	B/Nevada/26/2024	HA: EPI3267105<br>NA: EPI3267104	Southern Nevada Public Health Lab

**B/Victoria**	B/Wisconsin/06/2024	HA: EPI3070719<br>NA: EPI3070715	Wisconsin State Laboratory of Hygiene
	B/HongKong/228/2024	HA: EPI3834794<br>NA: EPI3834790	Government Virus Unit

Table S3. HA and HAI antibody titers

Subtype	Viruses	Clade	HA titer	HAI titer
H1N1pdm09	A/Michigan/45/2015	6B.1	/	640
	A/shenzhen/1/2019	6B.1A.7	32	640
	A/shenzhen/2/2019	6B.1A.5a	32	640
	A/shenzhen/4/2019	6B.1A.5a	64	1280
	A/shenzhen/5/2019	6B.1A.5a	16	640
	A/shenzhen/6/2019	6B.1A.5a	32	1280
	A/shenzhen/8/2019	6B.1A.7	16	320
	A/shenzhen/13/2019	6B.1A.5a	32	640
	A/shenzhen/15/2019	6B.1A.5a	32	1280
	A/Victoria/2570/2019	6B.1A.5a.2	/	2560
	A/shenzhen/2/2023	6B.1A.5a.2a	8	640
	A/shenzhen/3/2023	6B.1A.5a.2a	16	640
	A/shenzhen/5/2023	6B.1A.5a.2a	32	640
	A/shenzhen/7/2023	6B.1A.5a.2a	8	640
	A/shenzhen/9/2023	6B.1A.5a.2a	64	640
	A/shenzhen/11/2023	6B.1A.5a.2a	32	1280
	A/shenzhen/12/2023	6B.1A.5a.2a	16	1280
	A/shenzhen/13/2023	6B.1A.5a.2a	16	1280
	A/shenzhen/16/2023	6B.1A.5a.2a	8	640
	A/Wisconsin/67/2022	6B.1A.5a.2a.1	/	1280
	A/shenzhen/1/2024	6B.1A.5a.2a	32	320
	A/shenzhen/7/2024	6B.1A.5a.2a	256	2560
	A/shenzhen/11/2024	6B.1A.5a.2a	64	640
	A/shenzhen/17/2024	6B.1A.5a.2a	128	2560
	A/shenzhen/21/2024	6B.1A.5a.2a	64	640
H3N2	A/Singapore/INFIMH-16-0019/2016	3C.2a1	/	1280
	A/shenzhen/3/2019	3C.2a1b.1b	16	80
	A/shenzhen/4/2019	3C.2a1b.1b	16	160
	A/shenzhen/8/2019	3C.2a1b.2	32	640
	A/shenzhen/11/2019	3C.2a1b.1b	16	320
	A/Cambodia/e0826360/2020	3C.2a1b.2a.1a	/	640
	A/shenzhen/1/2022	3C.2a1b.2a.1a.1	8	160
	A/shenzhen/2/2022	3C.2a1b.2a.1a.1	8	160
	A/shenzhen/8/2022	3C.2a1b.2a.1a.1	8	320
	A/shenzhen/9/2022	3C.2a1b.2a.1a.1	8	320
	A/shenzhen/10/2022	3C.2a1b.2a.1a.1	8	160
	A/shenzhen/20/2022	3C.2a1b.2a.1a.1	16	320
	A/shenzhen/21/2022	3C.2a1b.2a.1a.1	8	320
	A/Darwin/6/2021	3C.2a1b.2a.2a	/	320
	A/shenzhen/1/2023	3C.2a1b.2a.2a.3a.1	128	320

	A/shenzhen/2/2023	3C.2a1b.2a.2a.3a.1	32	640
H3N2	A/shenzhen/4/2023	3C.2a1b.2a.2a.3a.1	8	320
	A/shenzhen/9/2023	3C.2a1b.2a.2a.3a.1	64	1280
	A/shenzhen/11/2023	3C.2a1b.2a.2a.3a.1	8	640
	A/Darwin6/2021	3C.2a1b.2a.2a	/	320
	A/shenzhen/5/2024	3C.2a1b.2a.2a.3a.1	32	640
	A/shenzhen/8/2024	3C.2a1b.2a.2a.3a.1	8	640
	A/shenzhen/12/2024	3C.2a1b.2a.2a.3a.1	64	320
	A/shenzhen/18/2024	3C.2a1b.2a.2a.3a.1	32	320
	A/shenzhen/23/2024	3C.2a1b.2a.2a.3a.1	32	640
B/Victoria	B/Colorado/06/2017	V1A.1	/	80
	B/shenzhen/1/2019	V1A.3	64	40
	B/shenzhen/2/2019	V1A.3	32	40
	B/shenzhen/3/2019	V1A.3	64	40
	B/shenzhen/6/2019	V1A.3	64	80
	B/shenzhen/7/2019	V1A.3	64	80
	B/shenzhen/8/2019	V1A.3	32	160
	B/shenzhen/9/2019	V1A.3	64	80
	B/shenzhen/10/2019	V1A.3	16	40
	B/Austria/1359417/2021	V1A.3a.2	/	640
	B/shenzhen/2/2023	V1A.3a.2	16	1280
	B/shenzhen/4/2023	V1A.3a.2	256	2560
	B/shenzhen/5/2023	V1A.3a.2	16	1280
	B/shenzhen/7/2023	V1A.3a.2	128	1280
	B/shenzhen/1/2024	V1A.3a.2	64	160
	B/shenzhen/4/2024	V1A.3a.2	128	160
	B/shenzhen/9/2024	V1A.3a.2	64	160
	B/shenzhen/14/2024	V1A.3a.2	128	320
